# SUMO: an R package for simulating multi-omics data for methods development and testing

**DOI:** 10.1093/bioadv/vbaf264

**Published:** 2025-10-22

**Authors:** Bernard Isekah Osang’ir, Surya Gupta, Ziv Shkedy, Jürgen Claesen

**Affiliations:** Microbiology Unit, Nuclear Medical Applications, Belgian Nuclear Research Centre, SCK CEN, Boeretang 190, Mol, 2400, Belgium; Data Science Institute, Hasselt University, Agoralaan, Diepenbeek, 3590, Belgium; Microbiology Unit, Nuclear Medical Applications, Belgian Nuclear Research Centre, SCK CEN, Boeretang 190, Mol, 2400, Belgium; Data Science Institute, Hasselt University, Agoralaan, Diepenbeek, 3590, Belgium; Data Science Institute, Hasselt University, Agoralaan, Diepenbeek, 3590, Belgium; Department of Epidemiology and Data Science, Amsterdam UMC, Amsterdam, 1081 HV, The Netherlands

## Abstract

**Motivation:**

Insights from integrative multi-omics analyses have fueled demand for innovative computational methods and tools in multi-omics research. However, the scarcity of multi-omics datasets with user-defined signal structures hinders the evaluation of these newly developed tools. SUMO (SimUlating Multi-Omics), an open-source R package, was developed to address this gap by enabling the generation of high-quality factor analysis-based datasets with full control over the dataset’s structure such as latent structures, noise, and complexity. Users can configure datasets with distinct and/or shared non-overlapping latent factors, enabling flexible and precise control over the signal structures. Consequently, SUMO allows reproducible testing and validation of methods, fostering methodological innovation.

**Availability and implementation:**

The SUMO R package is freely available and accessible on the Comprehensive R Archive Network https://doi.org/10.32614/CRAN.package.SUMO and on GitHub https://github.com/lucp12891/SUMO.git under CC-BY 4.0 license.

## 1 Introduction

The growing impact of multi-omics analysis has prompted a surge in experiments, raising both the complexity and the scale of integrative analyses. These developments pose significant challenges for existing multi-omics tools while simultaneously creating opportunities for innovative methodological advancements. To foster innovation in method development, it is crucial to systematically compare and validate newly developed multi-omics integration tools with existing methods. However, the limited availability of curated benchmark datasets with known signals for comparative evaluation of these tools remains a bottleneck. Furthermore, the availability of these datasets establishes a structured simulation environment that enables the systematic generation of diverse, multi-faceted datasets, providing a rigorous foundation for benchmarking, stress-testing, and comparative evaluation of these tools.

To address this, tools such as MOSim (Monzó *et al.* 2018), InterSIM (Chalise *et al.* 2016), OmicsSIMPLA (Chung and Kang 2019), and sismonr (Angelin-Bonnet *et al.* 2020) have emerged. Additionally, scDesign3 (Song *et al.* 2024) and scMultiSim (Li *et al.* 2025) have been developed for single-cell-oriented simulation frameworks. However, these multi-omics data simulators are not tailored for FA-based benchmarking in bulk omics. This lack of well-characterized simulation frameworks with clearly defined signal structures continue to hampers the development, validation, and rigorous evaluation of FA-based approaches for multi-omics integration. SUMO fills this gap by simulating datasets with explicit latent factors and traceable ground truth, enabling robust evaluation of FA-driven methods.

## 2 SUMO: an R package

Here, we present SUMO, an R package for simulating high-quality FA-based multi-omics datasets, granting users maximum control over noise levels, signal structures, and customizable dataset features for robust and reproducible benchmarking. Such flexibility and user-definability is crucial for rigorous and unbiased evaluation of tools, advancing the reliability and reproducibility, and broader adoption of multi-omics analysis methods and pipelines.

SUMO uses a generative factor model approach to generate multi-omics datasets by inverting the conventional FA-based model. For data analysis, standard matrix factorization methods typically decompose the observed data to infer lower-dimensional representations comprising latent factors with corresponding feature and sample contributions. SUMO reverses this process by starting with predefined latent structures and constructs datasets from these factors, incorporating their associated feature contributions (weights/loadings), sample contributions (factor scores), and user-defined, data-specific background noise. This methodology directly aligns with widely used FA-based approaches for multi-omics integration, such as Multi-Omics Factor Analysis, MOFA (Argelaguet *et al.* 2018), Group Factor Analysis, GFA (Klami *et al.* 2015), and Factor Analysis for Biclustering Acquisition, FABIA (Hochreiter *et al.* 2010, Kasim *et al.* 2016). In this context, SUMO is uniquely well suited for benchmarking FA-based methods, providing a critical bridge between methodological development and real-world application.

### 2.1 Generative factor model formulation

The core of SUMO’s data generation framework is a linear generative factor model, formulated in Equation (1), which decomposes each *m *×* n* data matrix (**X**), with *m* features and *n* samples:


(1) $$X=\Lambda^{T}\Gamma +Ε\;$$

where **Λ** is a *k *×* m* loading matrix representing the contribution of *m* features to *k* latent factors. Each row in **Λ**, denoted as λ_*i*_ (with *i *= 1,…,*k*), corresponds to the loading vector of the *i*th latent factor. **Γ** is a *k *×* n* latent factor matrix encoding the influence of each latent factor across *n* samples. Each column in **Γ**, denoted as γ_*j*_ (with *j *= 1,…,*n*), represents the factor scores for the *j*th sample. Residual variation (background noise) is modeled by **E**, an *m *×* n* matrix with entries drawn from a normal distribution with mean zero and constant variance σ^2^. Consequently, each element/entry of the simulated data, x_*ij*_, can be expressed as:


(2) $$x_{ij}=\lambda_{i}^{T}\gamma +\varepsilon_{ij}\;\mathrm{or}\;\mathrm{equivalently},x_{ij}={µ}_{ij}+\varepsilon_{ij}$$

where λ_*i*_ and γ_*j*_ represent the feature- and sample-specific contributions, respectively and ɛ_*ij*_ is the *ij*th entry of **E**.

**Figure 1.**
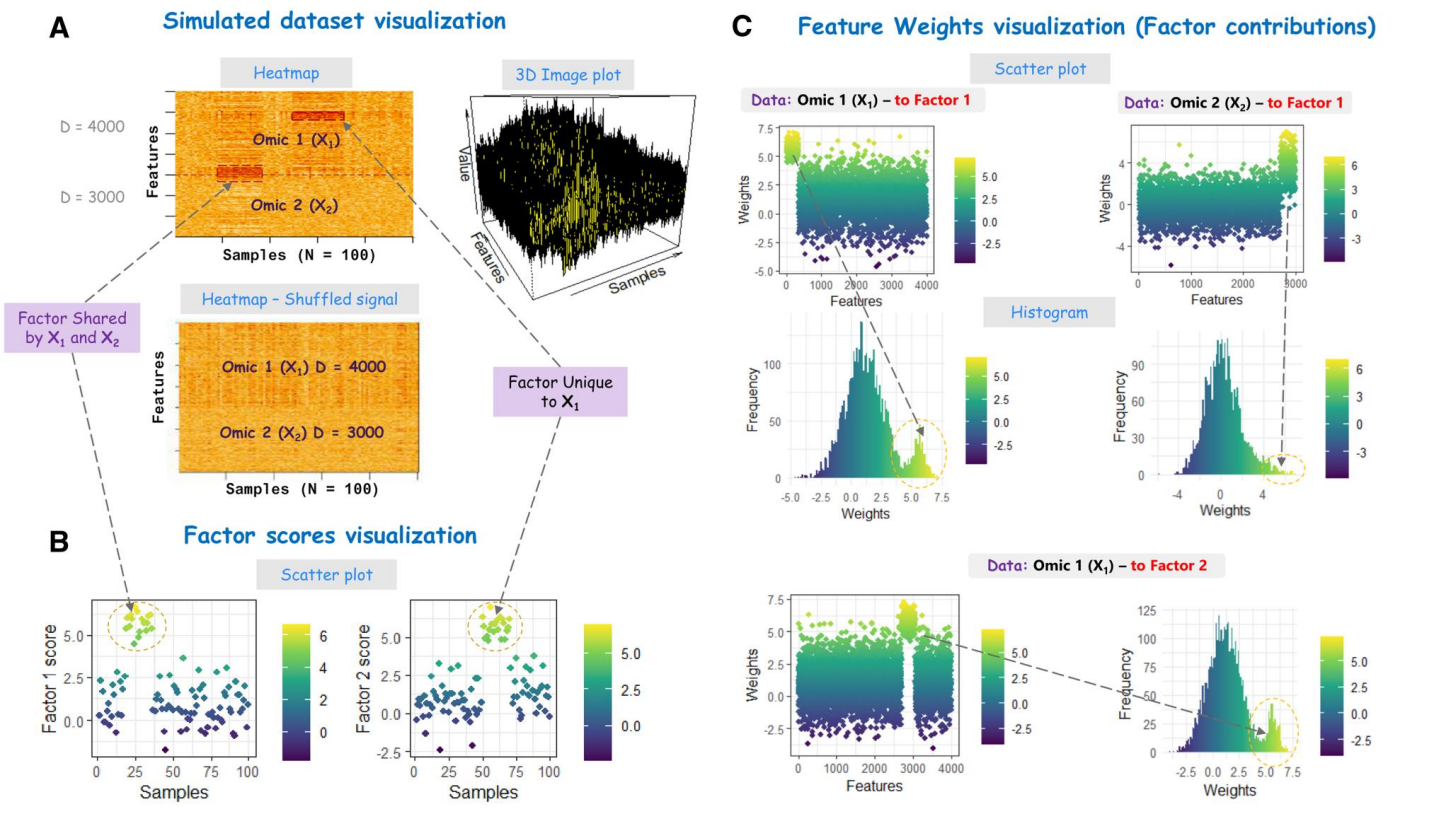
Visualization of simulated multi-omics datasets from SUMO illustrating latent factor and feature loadings/weights: (A) Heatmap and 3D surface plots of datasets with two user-defined latent factors: Factor 1 [shared across Omic 1 (**X_1_**) and Omic 2 (**X_2_**) and Factor 2 (specific to Omic 1 (**X_1_**)]. The heatmap includes an additional visualization with permuted signals to contrast structured and permuted signal patterns. (B) Scatter plots of factor scores, showing sample-level contributions to the shared (Factor 1) and unique (Factor 2) latent factors. (C) Scatter and histogram plots of feature loadings showing the distribution of feature contributions across both datasets, highlighting differences between features driven by shared versus unique factors and the influence of noise.

The model formulated in Equations (1) and (2) is defined for a single omics dataset. In the case of multi-omics data, with *m *≥ 2 data matrices, where each **X_*I*_** represents a distinct *m_I_*×*n* omic layer with *m_I_* features measured across a shared set of *n* biological samples. For instance, when *m *= 2, **X_1_** and **X_2_** may correspond to transcriptomics and proteomics data, respectively. The model in Equation (1) can then be generalized as:


(3) $$X_{I}=\Lambda_{I}^{T}\Gamma +E_{I\mathrm{for}\;I\;=\;2,\ldots ,\;m}$$

where **Γ** is a *k × n* shared score matrix capturing the latent structure across samples, **Λ_*I*_** are *m_I_×k* data-specific loading matrices, and **E_*I*_** are *m_I_×n* data-specific noise matrices. This formulation allows each omics layer to express distinct feature-level contributions through **Λ_*I*_**, while enforcing a common latent representation across all samples via **Γ**. Extended model derivations are provided in Section 2, available as supplementary data at *Bioinformatics Advances* online. The data generation framework follows the algorithm described in Equations (i)–(xiii), with Fig. 1, available as supplementary data at *Bioinformatics Advances* online (Section 2) further illustrates the formation of latent factors and the overall generation process, visually summarizing the mathematical principles presented in the equations.

### 2.2 Latent factor(s) configuration and multi-omics structure

The current SUMO framework generates omics datasets with heterogenous feature spaces and dimensionalities, but sharing a common sample set, a fundamental requirement for integrative multi-omics analysis (Argelaguet *et al.* 2018). Within this framework, users can explicitly define latent factors through factor scores and feature loadings, thereby specifying how the signal is distributed across features and samples. These latent structures can be configured as (i) unique factors, restricted to a single omics layer; (ii) shared factors, spanning multiple omics layers; or (iii) mixed structures, combining shared and unique components to capture cross-omics interactions and layer-specific variation (Table 1, available as supplementary data at *Bioinformatics Advances* online). SUMO adopts an early integration strategy wherein co-occurring samples serve as anchors to link omics layers into a unified multidimensional matrix (Fig. 4, available as supplementary data at *Bioinformatics Advances* online). Each omics layer contributes a distinct dimension to the data space, preserving latent structure while enabling the exploration of both cross-layer dependencies and layer-specific variation [Equation (3) and Fig. 1, available as supplementary data at *Bioinformatics Advances* online]. Although the underlying model assumes linear relationships, potentially limiting the ability to capture complex nonlinear dependencies seen in experimental omics data, SUMO nevertheless provides a structured, transparent, and reproducible framework for benchmarking and advancing multi-omics integration methods, particularly those grounded in matrix factorization. Details on package installation, dependencies, and setup are provided in the Section 2.4, available as supplementary data at *Bioinformatics Advances* online and the accompanying vignette. SUMO offers a built-in visualization functionality, to support inspection of simulated datasets, latent factors (via factor scores), and feature contributions (via weights/loadings). These visualizations enable users to assess distributional properties, signal structures, and sample- and feature-level variability. Available options include heatmaps for structured and permuted signals, 3D representations of the latent space, scatter plots for examining factor scores and dataset-specific loadings, and histograms to identify extreme values of these components (see Fig. 1). Collectively, these features support quality control of the simulated datasets and ensure suitability for rigorous downstream analyses.

## 3 SUMO workflow

**Figure 2.**
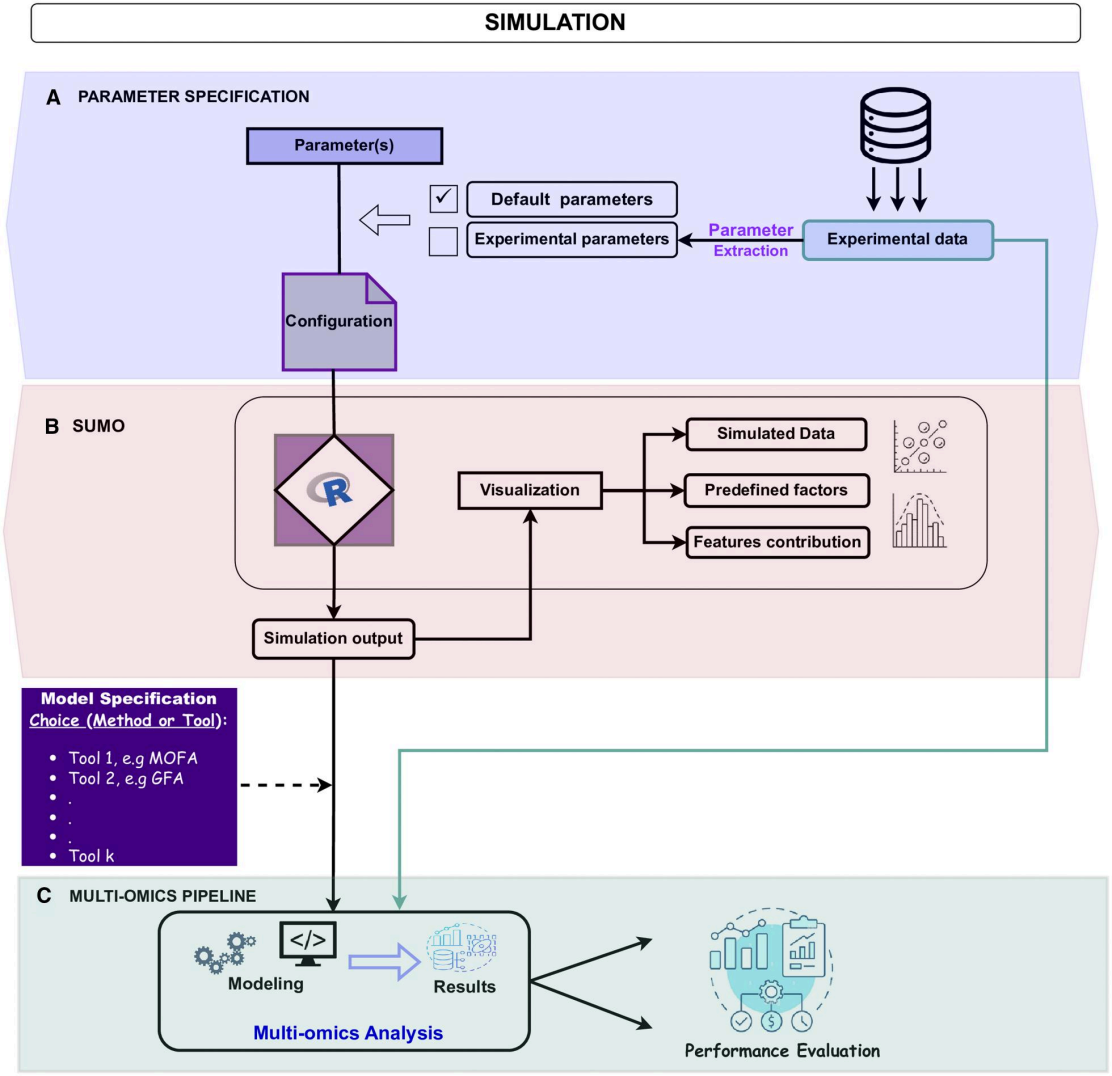
End-to-end SUMO data generation and benchmarking workflow. (A) Parameters are specified via a configuration function, using default settings or estimates derived from experimental data. (B) SUMO generates multi-omics datasets which are inspected using built-in visualizations prior to analysis. (C) The generated data are analyzed with multi-omics integration methods (e.g., MOFA), and performance is evaluated against the known ground truth.

Generation of multi-omics datasets with SUMO is illustrated in the workflow presented in Fig. 2. To illustrate its implementation of the SUMO framework, we constructed an example using an experimental chronic lymphocytic leukemia (CLL) dataset as a reference case study, previously explored by Argelaguet *et al.* (2018), and originally published in Dietrich *et al.* (2018). This multi-omics dataset was used to estimate input parameters such as signal characteristics and background noise, which informed the simulation settings. The resulting SUMO-generated dataset preserves the approximated key statistical and structural properties of the reference data such as feature-level and sample-level means, variances, and background noise, while allowing the controlled introduction of additional layers of variation through user-defined signals (for details refer to Section 3.4, available as supplementary data at *Bioinformatics Advances* online). As such, we do not expect SUMO-generated data to fully resemble real experimental datasets, since authentic biological variation cannot be completely simulated. But we do expect SUMO-generated data to fully capture the data structure defined by the user. Importantly, this is not a limitation: SUMO’s strength lies in enabling users to inject known signals and systematically track whether they are recovered, thereby supporting rigorous benchmarking of analytical tools. Accordingly, SUMO is not intended to replace real datasets; rather, it bridges method development and real-world application by enabling controllable, traceable ground-truth signals that experimental data do not provide. Although SUMO-generated datasets can serve as benchmarks for a wide range of computational models, either individually or in comparative evaluations, such assessments are beyond the scope of this manuscript. Here, we focus on demonstrating SUMO’s ability to generate structured, interpretable datasets suitable for testing FA-based multi-omics integration methods. A detailed description and results are provided in Section 3, available as supplementary data at *Bioinformatics Advances* online.

### 3.1 Parameter specification and configuration

The first step in the SUMO workflow is model parameterization (Fig. 2A). In this stage, users can either apply SUMO’s default parameter settings, or derive parameters directly from experimental datasets (example of CLL data provided in the Section 3, available as supplementary data at *Bioinformatics Advances* online). Before initiating a simulation, configurations should be verified against the specifications provided in the SUMO user manual. As an illustration of how SUMO can be calibrated from real data, we generated datasets and compared them with the CLL dataset as a reference. These comparisons show that SUMO dataset preserves expected variance structures and, although not specifically designed for clustering or correlation analyses, also maintains comparable patterns, underscoring its suitability for benchmarking (Sections 3.3 and 3.4, available as supplementary data at *Bioinformatics Advances* online). To further provide support to both expert and non-expert users, SUMO includes a vignette with detailed explanation of each parameter, and an example of simulation settings for method evaluation. This design ensures accessibility for both expert and non-expert users, enabling the generation of datasets tailored to their diverse analytical objectives and benchmarking needs.

### 3.2 Data generation and visualization

The simulated data is generated by SUMO using the user-defined parameters. The built-in visualization functions (Fig. 2B) can be further used to inspect simulated datasets distributions (heatmaps, 3-D image plot; Fig. 1A), to explore the underlying latent factor structures (scatter plot; Fig. 1B), and to assess feature contributions (scatter plot and histogram; Fig. 1C). These inspections provide valuable insights into the structure of the generated dataset, and the potential for accurate recovery of true signal. For example in Fig. 1, the clear separation between the simulated signal and background noise, suggests that the true signal will be easily recovered.

### 3.3 Performance evaluation environment

The generated simulated dataset can be further analyzed through a multi-omics analysis pipeline, such as MOFA (Fig. 2C). The resulting outputs can then be systematically evaluated for performance against the predefined signals embedded during simulation. SUMO offers explicit control over latent factor structures and full traceability of sample- and feature-level contributions, thereby enabling rigorous assessment of how effectively computational methods recover the underlying true signals. As discussed in Section 3, available as supplementary data at *Bioinformatics Advances* online, SUMO allows seamless integration of multi-omics analysis methods, such as MOFA (see Section 3, available as supplementary data at *Bioinformatics Advances* online for details). Beyond evaluating latent factor recovery, SUMO-generated datasets support evaluation across performance metrics including accuracy, sensitivity, specificity, and robustness to noise. For instance, the impact of noise variation controlled via the signal-to-noise ratio (SNR) on model performance is illustrated in Figs 6a and b, available as supplementary data at *Bioinformatics Advances* online (Section 3). Finally, the pipeline outlined in Fig. 2, available as supplementary data at *Bioinformatics Advances* online is a proof-of-concept workflow developed in-house, which serves to demonstrate how SUMO can be integrated into diverse analytical pipelines to objectively benchmark and compare multi-omics integration tools in a flexible and reproducible manner.

## 4 Conclusions

SUMO is a flexible and extensible R package designed to simulate structured multi-omics datasets, offering precise control over latent structures, noise levels, and overall data complexity. Beyond its flexibility, it establishes a reproducible and standardized environment for developing and validating computational methods. Its compatibility with existing pipelines ensures seamless integration into established workflows, accelerating efficient method development and evaluation. By offering transparent benchmarking and optimization, SUMO provides a critical foundation for the advancement of computational innovation in multi-omics research.

## Supplementary Material

vbaf264_Supplementary_Data

## Data Availability

The data underlying this article are available in the SUMO GitHub repository at https://github.com/lucp12891/SUMO and in the Comprehensive R Archive Network (CRAN) at https://doi.org/10.32614/CRAN.package.SUMO. The package (current version 1.2.2) includes all code, example test data, and documentation needed to reproduce the results presented in this manuscript.
