# Supplementary material for: SUMO: an R package for simulating multi-omics data for methods development and testing
Source: Bioinform Adv. 2025 Oct 22;5(1):vbaf264. doi: 10.1093/bioadv/vbaf264 (PMC12630132; doi:10.1093/bioadv/vbaf264)
Supplement: vbaf264_Supplementary_Data [file vbaf264_supplementary_data.pdf]

Supplementary Information

SUMO: An R Package for Simulating Multi-Omics Datasets for Method Development and Testing

Bernard Isekah Osang'ir[1,2], Surya Gupta[1] @, Ziv Shkedy[2], and Jürgen Claesen[2,3]

[1] SCK•CEN, Belgian Nuclear Research Centre, Mol, Belgium

[2] Hasselt University, Data Science Institute, Center for Statistics, Diepenbeek, Belgium

[3] Department of Epidemiology and Data Science, Amsterdam UMC, Amsterdam, The Netherlands

1 Introduction

This Supplementary Material provides a comprehensive overview of the SUMO R package, a simulation framework for generating multi-omics datasets tailored to method development and evaluation. Built on a flexible factorization-based model, SUMO allows users to define shared and unique latent structures across omics layers, creating a realistic yet controlled environment for benchmarking factor analysis (FA)-based multi-omics methods. The Supplement elaborates on the mathematical formulations, provides detailed installation and usage guidance, and illustrates practical applications through worked examples. It is intended as a technical companion to the main manuscript.

2 Package: Extended Methodology

This section provides a detailed account of the methodology used to generate simulated multi-omics datasets, complementing the description in the main paper. The data generation strategy is grounded in a factorization-based statistical model, offering a structured yet flexible framework to represent latent biological signals shared across omics layers. We begin by outlining the general structure of multi-omics data and then present the mathematical model underlying the simulation process in detail.

2.1 Representation of multi-omics data

Consider an experiment that generates multiple distinct omics datasets—for example, gene expression and protein expression. Each dataset can be represented as a data matrix, $\mathbf{X}$, with rows corresponding to measured features (e.g., genes or proteins) and columns to samples. If two datasets, $\mathbf{X}_1$ (e.g. RNA-seq) and $\mathbf{X}_2$ (e.g. proteomics) are derived from the same set of n samples then they can be represented mathematically as:

$$\mathbf{X_1} = \underset{m_1 \text{ features e.g. genes}}{\overset{n \text{ samples}}{\begin{bmatrix} x_{1,11} & x_{1,12} & \cdots & x_{1,1n} \\ x_{1,21} & x_{1,22} & \cdots & x_{1,2n} \\ \vdots & \vdots & \ddots & \vdots \\ x_{1,l1} & x_{1,l2} & \cdots & x_{1,ln} \end{bmatrix}}}, \quad \mathbf{X_2} = \underset{m_2 \text{ features e.g. proteins}}{\overset{n \text{ samples}}{\begin{bmatrix} x_{2,11} & x_{2,12} & \cdots & x_{2,1n} \\ x_{2,21} & x_{2,22} & \cdots & x_{2,2n} \\ \vdots & \vdots & \ddots & \vdots \\ x_{2,l1} & x_{2,l2} & \cdots & x_{2,ln} \end{bmatrix}}} \tag{i}$$

Each dataset may contain a different number of features, m_1 and m_2, respectively, yet both share the same set of samples, n. These distinct features correspond to different biological layers and often vary substantially in measurement scale and variance structure [Argelaguet et al., 2018].

2.2 Inversion of Matrix Factorization Model

To systematically generate multi-omics datasets, we assume that the observed data matrices are governed by a low-dimensional latent structure (factors). Without loss of generality, consider a dataset ($\mathbf{X}$) of dimension $m \times n$, where m denotes the number of measured features and n the number of samples. The generative process for this dataset can be formulated using a matrix factorization model as follows:

$$\mathbf{X} = \Lambda^{\intercal}\Gamma + \mathbf{E} \tag{ii}$$

where:

- Λ is a $k \times n$ is the loading matrix, representing the contribution of features to k latent factors. Each row λ_i (with $i = 1,...,k$), corresponding to the loading vectors, forming Λ can be written as $\Lambda = (\lambda_1, \lambda_2, \ldots, \lambda_k)$.
- Γ is a $k \times n$ is the latent factor matrix, encoding the influence of each latent factor across samples. Each column γ_j (with $j = 1,...,n$) represents the factor scores for a sample, forming $\Gamma = (\gamma_1, \gamma_2, \ldots, \gamma_n)$.

- **E** is a $m \times n$ residual noise matrix assume to follow a normal distribution with mean zero and constant variance, σ^2.

More explicitly, for any given measurement x_{ij} corresponding to the ith feature and jth sample, the factorization model in equation (ii) can be represented as:

$$x_{ij} = \lambda_i^{\mathsf{T}} \gamma_j + \varepsilon_{ij} \quad \text{which can be expressed further as} \quad x_{ij} = \mu_{ij} + \varepsilon_{ij} \tag{iii}$$

with

$$\mu_{ij} = \lambda_i^{\mathsf{T}} \gamma_j \tag{iv}$$

When considering a single latent factor $k = 1$, equation (iv) simplifies to:

$$\mu^{(1)} = \mu_{ij}^{(1)} = \lambda_i \gamma_j. \tag{v}$$

The model defined in Equations (ii)–(v) represents a single-omics dataset as a linear combination of latent factors and residual noise. Grounded in factor analysis (FA), this approach is widely used to uncover hidden sources of variation underlying the observed data. In a single omics layer, the data matrix is decomposed into the product of a loading matrix (feature contributions) and a score matrix (sample-level latent structure), with an added noise term capturing unexplained variation.

In the multi-omics setting, where multiple biological layers are profiled on the same set of samples, the single-omics FA model can be extended to represent both shared and layer-specific structures. For example, consider two omics layers—transcriptomics ($\mathbf{X}_1$) and proteomics ($\mathbf{X}_2$)—each expressed as a data matrix. The generalized multi-omics model can then be formulated as:

$$\begin{aligned} \mathbf{X}_1 &= \Lambda_1 \Gamma + \mathbf{E}_1 \\ \mathbf{X}_2 &= \Lambda_2 \Gamma + \mathbf{E}_2 \end{aligned} \tag{vi}$$

In this formulation, $\mathbf{X}_1$ and $\mathbf{X}_2$ are real-valued matrices of dimensions $m_1 \times n$ and $m_2 \times n$, respectively. Rows correspond to features (e.g., genes or proteins) and columns to biological samples. The shared sample dimension (n) provides the basis for integration across omics layers. The model is defined by three key components:

- **Shared latent factors** (Γ, of size $k \times n$) represent low-dimensional sample-level variation that is common across all omics layers, forming the underlying structure that drives the observed measurements.
- **Omics-specific loading matrices** (Λ_1, Λ_2) of dimensions $m_1 \times k$ and $m_2 \times k$, respectively, map features within each omics layer to the shared latent factors. This formulation enables each layer to express the common latent structure in a biologically distinct manner, reflecting differences in signal manifestation across omics types.
- **Data-specific noise matrices** ($\mathbf{E}_1$, $\mathbf{E}_2$) of size $m_1 \times n$ and $m_2 \times n$, respectively, capture residual variation and measurement noise unique to each dataset.

The two datasets can also be conceptualized as components of a unified multi-omics matrix. Depending on the implementation, they may either be concatenated row-wise or represented as a collection of matrices. Their joint structure is illustrated in Equation (vii).

$$\mathbf{X} = \begin{array}{c} \\ m_1 \text{ genes} \\ \\ m_2 \text{ proteins} \end{array} \overset{n \text{ samples}}{\begin{bmatrix} x_{1,11} & x_{1,12} & \cdots & x_{1,1n} \\ x_{1,21} & x_{1,22} & \cdots & x_{1,2n} \\ \vdots & \vdots & \ddots & \vdots \\ x_{1,m_1 1} & x_{1,m_1 2} & \cdots & x_{1,m_1 n} \\ \\ x_{2,11} & x_{2,12} & \cdots & x_{2,1n} \\ x_{2,21} & x_{2,22} & \cdots & x_{2,2n} \\ \vdots & \vdots & \ddots & \vdots \\ x_{2,m_2 1} & x_{2,m_2 2} & \cdots & x_{2,m_2 n} \end{bmatrix}} \tag{vii}$$

This framework naturally extends to $m \geq 2$ omics datasets. For each omics layer $I = 1, \ldots, m$, we define a data matrix $\mathbf{X}_I$ of dimension $m_I \times n$, where m_I denotes the number of features in layer I, and all layers share the same n samples. The generalized multi-omics FA model is expressed as:

$$\mathbf{X}_I = \Lambda_I \Gamma + \mathbf{E}_I, \quad \text{for } I = 1, \ldots, m \tag{viii}$$

where:

- Γ is the shared score matrix ($k \times n$), representing latent structure across samples.
- Λ_I is the loading matrix ($m_I \times k$) for omics layer I, mapping features to latent dimensions.
- $\mathbf{E}_I$ is the noise matrix ($m_I \times n$), modeling residual and measurement variation.

This flexible formulation allows FA-based integration methods to capture both global patterns (shared across layers) and omics-specific signals, supporting deeper biological insights and more interpretable data decomposition in multi-omics analysis.

2.3 Specification of signal structures

For dataset generation, it is crucial to explicitly define the latent factor structure in terms of sample scores and feature loadings (weights). Suppose predefined signals correspond to specific biological conditions or subsets of interest; in that case, the feature loading vectors (λ_i) and factor scores (γ_j) can be directly specified as:

$$\lambda_i = \begin{cases} \lambda_i \sim N(\mu_{\lambda_i}, \sigma^2_{\lambda_i}), & \text{if feature } i \text{ belongs to the signal subset} \\ 0, & \text{otherwise (uninformative feature)} \end{cases}$$

$$\gamma_j = \begin{cases} \gamma_j \sim N(\mu_{\gamma_j}, \sigma^2_{\gamma_j}), & \text{if sample } j \text{ belongs to the signal subset} \\ 0, & \text{otherwise (uninformative sample)} \end{cases} \tag{ix}$$

Therefore, the signal structure μ_{ij} within the data can be explicitly defined as:

$$\mu_{ij} = \begin{cases} \vartheta, & \text{if both feature } i \text{ and sample } j \text{ belong to the predefined signal subset} \\ 0, & \text{otherwise (uninformative entry)} \end{cases} \tag{x}$$

Here, ϑ represents the signal contribution associated with the predefined subset. The residual noise term ε_{ij} is modeled as Gaussian:

$$\varepsilon_{ij} \sim N(0, \sigma^2_{\varepsilon}). \tag{xi}$$

Equations (ix)–(xi) establish a probabilistic framework for generating sample and feature components. By systematically varying parameters such as signal strength, variance, and feature subsets, the simulated datasets capture layered complexities that approximate those observed in multi-omics experiments.

The model in Equation (ix) assumes that $\mu_{ij} = \vartheta$, where ϑ denotes the signal contribution, if both the ith feature and the jth sample belong to the predefined signal subset; otherwise, $\mu_{ij} = 0$. his formulation ensures that signal is expressed only when specific features and samples co-occur within the defined subset, thereby representing the latent factor.

Equation (iii) illustrates how individual matrix entries are derived by combining factor scores and feature

loadings with an added noise term. Figure S1a demonstrates this process for a single-factor scenario, where the product of feature loadings and factor scores yields a matrix representing one latent factor (Equations iii–viii). Figure S1b extends this principle to two factors within the same dataset, showing that each factor (1 and 2) is constructed independently using the same procedure outlined for the single-factor case.

To generalize, when multiple latent factors are present in a single dataset $\mathbf{X}$, the overall signal μ can be expressed as the sum of individual factor contributions (Figure S1a), formulated in Equations (xii) and (xiii):

$$\begin{aligned}
\mu &= \mu^{(1)} + \mu^{(2)} + \cdots + \mu^{(K)} \\
&= \sum_{k=1}^{K} \mu^{(k)}, \quad \text{where} \quad \mu^{(k)} = \lambda_k \gamma_k^\top \\
&= \lambda_1 \gamma_1^\top + \lambda_2 \gamma_2^\top + \cdots + \lambda_K \gamma_K^\top \\
&= \sum_{k=1}^{K} \lambda_k \gamma_k^\top
\end{aligned} \tag{xii}$$

This cumulative formulation accommodates dataset complexity by reflecting the multi-factor structure inherent in real data. Incorporating noise, the full model (Equation ii) can be expressed as:

$$\begin{aligned}
\mathbf{X} &= \mu + \varepsilon \\
&= \sum_{k=1}^{K} \mu^{(k)} + \varepsilon \\
&= \sum_{k=1}^{K} \lambda_k \gamma_k^\top + \varepsilon,
\end{aligned} \tag{xiii}$$

where $\varepsilon \sim N(0, \sigma_\varepsilon^2)$.

A pivotal aspect of our simulation framework is the reversal of the matrix factorization process described in Equations (ii) and (iii). Using the parameterizations in Equations (iv)–(xiii), the original dataset matrix $\mathbf{X}$ and each individual measurement X_{ij}, can be systematically reconstructed.

The simulation framework, illustrated in Figures S1a and S1b, extends the data generation process from a single dataset source to multiple sources (Equations iii–xiii). In this example, two datasets are constructed: $\mathbf{X_1}$, containing two latent factors, and $\mathbf{X_2}$, containing one. For each dataset, feature loadings and factor scores are combined through vector multiplication, and noise is added to simulate variability and previously discussed. These datasets are then concatenated, creating a comprehensive joint dataset that preserves shared factors across sources and unique factor in the first dataset.

Although distinct and varying in features, these datasets share a common set of n co-occurring subjects or samples, enabling integration [Argelaguet et al., 2018]. The SUMO framework leverages this commonality to concatenate the datasets, $\mathbf{X_1}$ and $\mathbf{X_2}$, into a single multidimensional matrix. This joint representation preserves latent factors and noise patterns, capturing complex interrelationships across datasets. As a result, users can analyze both within- and cross-layer relationships.

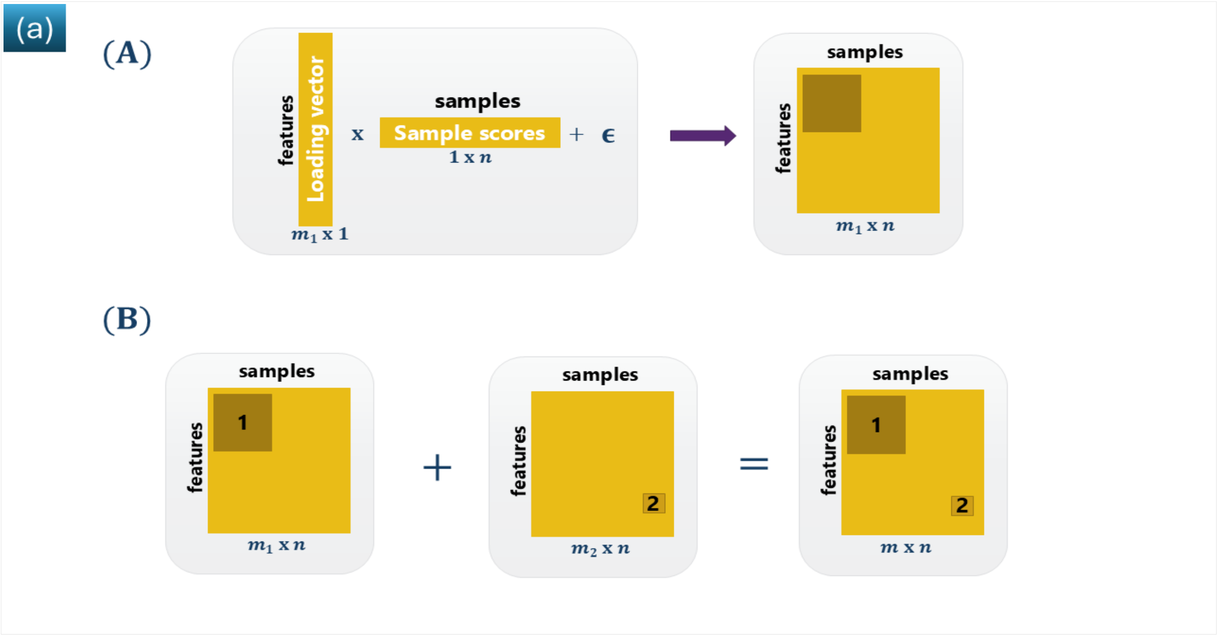

(a) Panels show factor formation through feature loading and score multiplication; Figure (A) illustrates the multiplication of feature loadings and factor scores, resulting in a matrix with a single factor. Figure (B) demonstrates a single dataset source with two factors, where each factor (1) and (2) is constructed in the same way as shown in Figure (A).

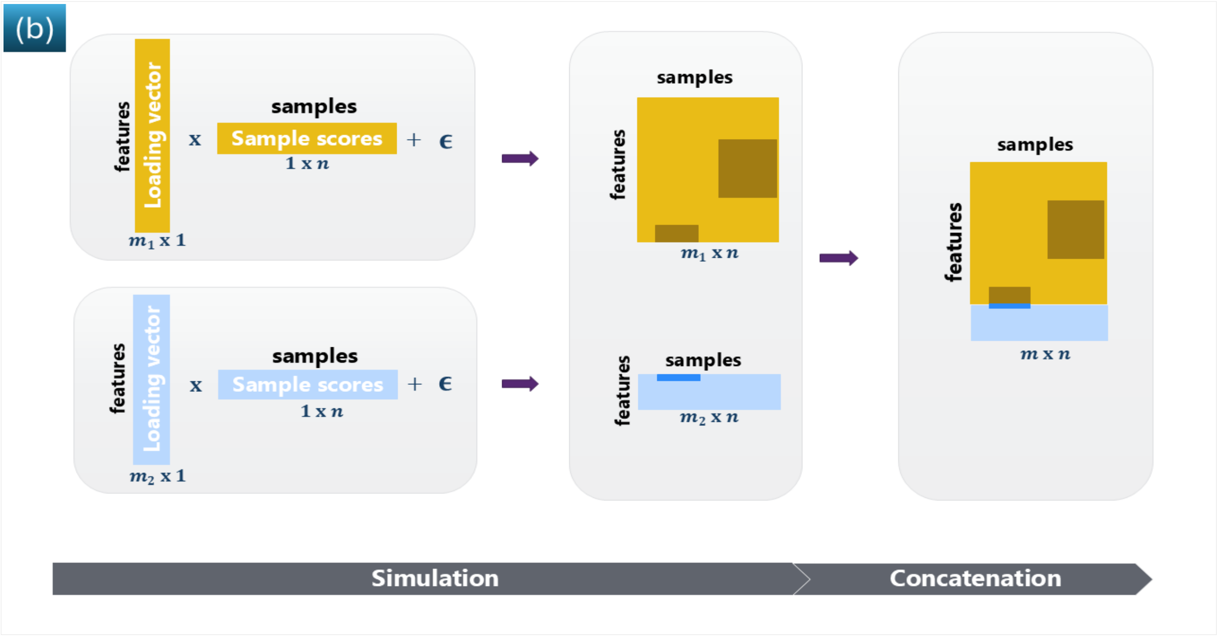

(b) Panel illustrates the simulation workflow and merging factors. ε is the residual noise matrix; The illustration of the simulation workflow. Vector multiplication is used to generate two datasets: one containing two factors and the other containing a single factor. Merging two datasets into a single multi-dimensional data with a shared factor and a unique factor.

Figure S1: Illustration of factor formation and simulation process: (a) Factor formation through feature loading and score multiplication, demonstrating single- and two-factor structures. (b) Simulation workflow showing dataset generation, factor merging, and the incorporation of data-specific residual noise (ε).

2.4 Package Installation and set-up

The SUMO package is freely available via the Comprehensive R Archive Network (CRAN; https://doi.org/10.32614/CRAN.package.SUMO) and on GitHub (https://github.com/lucp12891/SUMO.git). The package is actively maintained by SCK•CEN in collaboration with Hasselt University, ensuring long-term accessibility and support. SUMO is distributed under a CC-BY-NC 4.0 license, which permits users to freely download, share, and adapt the software for non-commercial purposes, provided appropriate attribution is given. This licensing framework promotes broad adoption within the academic community, while fostering transparency, reproducibility, and methodological innovation in multi-omics research

2.4.1 *R Environment Setup*

To ensure reproducibility and ease of use, it is important to configure the R environment correctly before running SUMO. The package was developed and tested under R version 4.3.3, and we recommend using this version or later for optimal compatibility and performance. While validation has been performed primarily on Windows 10 and later, SUMO can also be deployed on macOS and most modern Linux distributions.

SUMO relies on several R packages for its full functionality. Core dependencies include `ggplot2` [Wickham et al., 2016], `gridExtra` [Auguie et al., 2017], `rlang`, `stats`, `graphics`, and `utils`, which support visualization, data handling, and base statistical functionality. In addition, SUMO integrates seamlessly with tidy data workflows through `dplyr`, `readr`, `readxl`, `stringr`, `magrittr`, and `data.table`, while high-quality reporting and export features are enabled via `officer`, `rvg`, and `systemfonts`. Advanced functionality for method benchmarking leverages `MOFA2`, `MOFAdata`, and `fabia`, as well as `basilisk` for environment management.

For reproducible testing, SUMO employs the `testthat` framework (version 3.0.0 or later; Config/testthat/edition: 3). Together, these dependencies ensure that SUMO can simulate, visualize, and benchmark multi-omics datasets within a robust and reproducible computational environment. Detailed installation

instructions for these packages are provided in Section 2.4.2.

To maximize accessibility, SUMO is accompanied by a comprehensive vignette that include tutorials, bundled test datasets, and plain-language parameter explanations. These resources are designed to support both expert and non-expert users in confidently simulating multi-omics datasets and linking parameter choices to evaluation goals. The vignette has been published online at RPubs (RPubs – SUMO: Simulation Utilities for Multi-Omics Data) and is also available in the SUMO GitHub repository (SUMO/-SUMO_Simulation Utilities for Multi-Omics Data.pdf). In addition, detailed documentation of all input and output parameters is provided on the package manual hosted on CRAN R-Universe (https://cran.r-universe.dev/SUMO/doc/manual.html).

Apart from the demonstration function in SUMO—which uses the Chronic Lymphocytic Leukemia dataset from Argelaguet et al. [2023] and therefore requires the `MOFA2` and `MOFAdata` packages—the package requires no external datasets to function. Examples illustrating how to use the different functionalities are included in both the vignette and the user documentation, ensuring that the framework can be readily adopted and reproduced within diverse analytical settings.

2.4.2 *Installation of dependencies*

To ensure full functionality of SUMO, users must install a set of R packages that provide essential statistical, visualization, and benchmarking capabilities. The dependencies fall into three categories: (i) core visualization and statistical libraries, (ii) multi-omics benchmarking packages, and (iii) system-level and reporting utilities. The instructions below summarize their installation.

(i) **SUMO**: The core package introduced in this application note. SUMO can be directly installed from CRAN.

```r
install.packages("SUMO")

```

(ii) **Core visualization and statistical dependencies**: `ggplot2` [Wickham et al., 2016] is essential for producing all plots in this document, while `gridExtra` [Auguie et al., 2017], `grid`, and `systemfonts`

support flexible layout management and high-quality graphics rendering. The `stats` [R Core Team, 2013] and `graphics` [R Core Team, 2013] packages provide statistical methods and base plotting functions, while `rlang`, `utils`, `dplyr`, `readr`, `readxl`, `stringr`, `data.table`, and `magrittr` extend the package with tidyverse-style data manipulation, I/O, and pipeline capabilities.

```
install.packages(c("ggplot2", "gridExtra", "grid", "systemfonts",
                   "stats", "graphics", "rlang", "utils", "dplyr",
                   "readr", "readxl", "stringr", "data.table", "
magrittr"))

```

(iii) **Multi-omics benchmarking and modeling dependencies**: SUMO interfaces with FA-based methods such as `MOFA2` [Argelaguet et al., 2018], its companion dataset package `MOFAdata`, and `fabia` [Hochreiter et al., 2010]. These allow demonstration and benchmarking of simulated data against established multi-omics methods. `basilisk` ensures compatibility of Python-based components within R.

```
if(!requireNamespace("BiocManager", quietly = TRUE))
  install.packages("BiocManager")
BiocManager::install("MOFA2")

install.packages("remotes")
remotes::install_github("bioFAM/MOFAdata")

install.packages("fabia")
install.packages("basilisk")

```

(iv) **Reporting and reproducibility utilities**: SUMO supports high-quality reporting and reproducible pipelines through packages such as `officer` (for automated Word and PowerPoint generation), `rvg` (for vector graphics), and `testthat` (for unit testing, edition 3).

```
install.packages(c("officer", "rvg", "testthat"))

```

Once installed, the packages can be loaded into the R session using the following command:

```r
library(SUMO)
library(ggplot2)
library(gridExtra)
library(grid)
library(systemfonts)
library(stats)
library(graphics)
library(rlang)
library(utils)
library(dplyr)
library(readr)
library(readxl)
library(stringr)
library(data.table)
library(magrittr)
library(MOFA2)
library(MOFAdata)
library(fabia)
library(basilisk)
library(officer)
library(rvg)
library(testthat)
```

2.5 SUMO Usage

SUMO is specifically designed to simulate multiple datasets that can be concatenated to form a comprehensive multi-omics data structure. The generated datasets reproduce several defining characteristics of biological data, thereby providing a controlled yet biologically plausible foundation for method development and evaluation. One of the most distinctive features of SUMO is its ability to generate both *unique* factors and *shared* factors across different dataset sources, making it a powerful tool for benchmarking integrative analysis methods.

Unique factors correspond to latent structures that are active exclusively within a single dataset source, thereby modeling modality-specific signals. In contrast, shared factors capture correlated latent structures that manifest across multiple datasets simultaneously, reflecting coordinated biological processes spanning omics layers [Brown et al., 2023]. By enabling this distinction, SUMO allows users to emulate both modality-specific and cross-modality dependencies, which are central to multi-omics integration studies.

The framework provides users with the flexibility to specify whether they require `single` or `multiple` latent factors, and whether these factors should be `unique` to one dataset, `shared` across datasets, or a combination of both. Specifically:

- **Single-factor datasets** can represent either a `unique` factor (specific to one dataset) or a `shared` factor (spanning multiple datasets).
- **Multiple-factor datasets** extend this principle to allow three possible configurations: (i) exclusively `unique` factors, (ii) exclusively `shared` factors, or (iii) `mixed` factors, where both unique and shared signals coexist.

The `mixed` configuration is particularly important, as it reflects the reality of multi-omics studies where some molecular mechanisms are dataset-specific, while others operate across multiple layers in a coordinated fashion. Table 1 provides a structured overview of these possible factor configurations. By supporting such flexible signal structures, SUMO enables systematic and reproducible evaluation of integrative methods under scenarios that approximate real-world biological heterogeneity.

Table 1: Summary of predefined factors

		Type of Factors	
		single factor	multiple factors
Factor(s)	Unique	✓	✓
	Shared	✓	✓
	Combined		✓

3 SUMO workflow

Simulation studies have long played a central role in computational biology, providing a rigorous and unbiased means of evaluating algorithmic performance under controlled and reproducible conditions. Unlike experimental datasets, which are often confounded by technical variability, missing values, and heterogeneous study designs, simulated datasets allow researchers to systematically manipulate key parameters—such as noise levels, signal strength, sample size, and dataset sparsity—to isolate their impact on method performance [Hasin et al., 2017]. This controlled setting ensures that methodological limitations can be diagnosed with precision, and that algorithmic robustness can be assessed before methods are applied to real-world data.

For integrative multi-omics analyses, where the interplay between unique and shared signals across modalities is often complex, such controlled evaluation becomes indispensable. Benchmarking in the absence of simulation may obscure whether performance differences arise from biological signal, data heterogeneity, or method assumptions. Therefore, carefully designed simulation frameworks are critical to establish both baseline expectations and upper performance bounds.

The SUMO R package directly addresses this need by providing a structured, flexible, and biologically informed framework for multi-omics simulation. SUMO enables the generation of datasets with explicitly defined latent structures, allowing researchers to fine-tune factor configurations, adjust signal-to-noise ratios, and explore scenarios ranging from simple single-factor datasets to complex mixed-factor landscapes. These simulated datasets serve as an initial testbed in which computational models can be systematically tuned, stress-tested, and benchmarked against known ground truth structures. In doing so, SUMO not only accelerates methodological development but also provides a robust validation layer prior to deploying integrative methods on real multi-omics datasets, where true signal structure remains unknown.

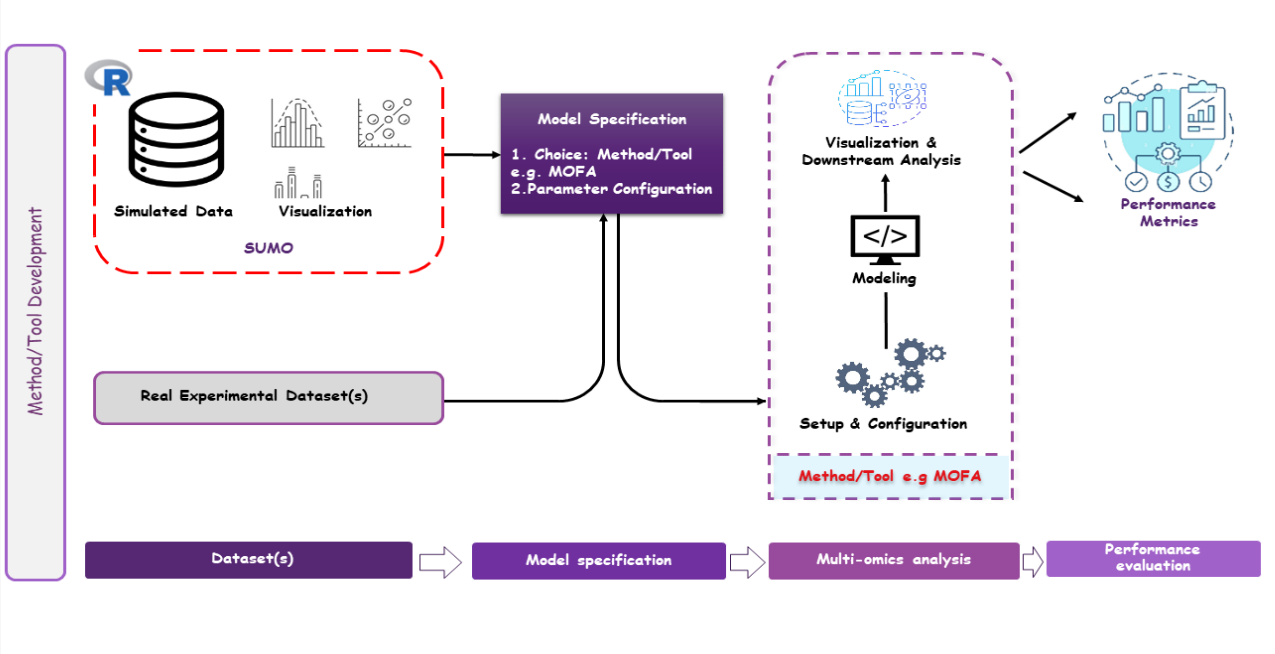

Figure S2: Workflow for benchmarking and evaluation of multi-omics methods

The conceptual workflow presented in Figure S2 illustrates the end-to-end pipeline from method development to performance evaluation, integrating both simulated and real-world datasets. This schematic is not only illustrative but also reflects the iterative strategy typically employed in practice, with sufficient flexibility to incorporate diverse computational tools at each stage.

A cornerstone of this process is the use of simulated datasets during the early phases of method evaluation—precisely where SUMO makes its most critical contribution. While the development phase (left section) generally encompasses algorithm design and parameter tuning within established frameworks (e.g., MOFA), the present work deliberately focuses on the subsequent stages: systematic testing and benchmarking. Before any method is applied to experimental multi-omics data, it is essential to ensure that its core functionality, stability, and scalability are validated under controlled conditions.

Simulated datasets provide the necessary clarity by offering ground truth structures against which model performance can be objectively assessed. They allow for rigorous interrogation of how algorithms respond to varying noise levels, factor configurations, and data complexities. SUMO directly facilitates this process by enabling the generation of realistic multi-omics datasets with tunable latent structures, thereby serving as a robust testbed for benchmarking integrative methods. In doing so, SUMO bridges

the critical gap between abstract algorithmic development and the complexities inherent to real-world biological datasets, ensuring that novel methodologies are stress-tested before deployment.

In the following section, we focus on the SUMO component (highlighted in red), examining its design, functionality, and role in generating datasets for integrative method validation. As outlined in Figure S3, the simulation process can be conceptualized in three sequential stages: parameter specification, dataset generation, and integration into multi-omics pipelines. Users first define the simulation settings, either using SUMO's default options or calibrating parameters from experimentally derived data; SUMO then generates structured datasets with controllable latent structures, including unique, shared, or mixed factors across omics layers; finally, these outputs can be directly incorporated into downstream workflows for benchmarking, stress-testing, and performance evaluation. This streamlined pipeline provides a reproducible and transparent pathway from parameter specification to method assessment, ensuring that simulated datasets function not merely as illustrative examples but as robust testbeds for advancing multi-omics integration methodologies.

3.1 Parameter Specification and Configuration

The utility and credibility of any simulation framework hinge on the precise specification of its input parameters, as these fundamentally determine both the structure and interpretability of the resulting datasets. Within SUMO, users are afforded fine-grained control over the simulation process through parameters that govern latent factor structures, feature loadings, sample scores, noise distributions, and patterns of factor sharing across omics layers. In practice, these settings dictate not only the type of signal to be introduced, but also its magnitude, the omics layers in which it manifests, and the extent to which it is obscured by noise. By enabling this level of configurability, SUMO ensures that simulated datasets can be tailored to reflect diverse experimental scenarios, ranging from simple single-factor designs to complex multi-factor architectures with overlapping signals. The following section provides a detailed exposition of these configurable parameters, highlighting their role in shaping the generated data and, ultimately, in supporting rigorous benchmarking of integrative analysis methods.

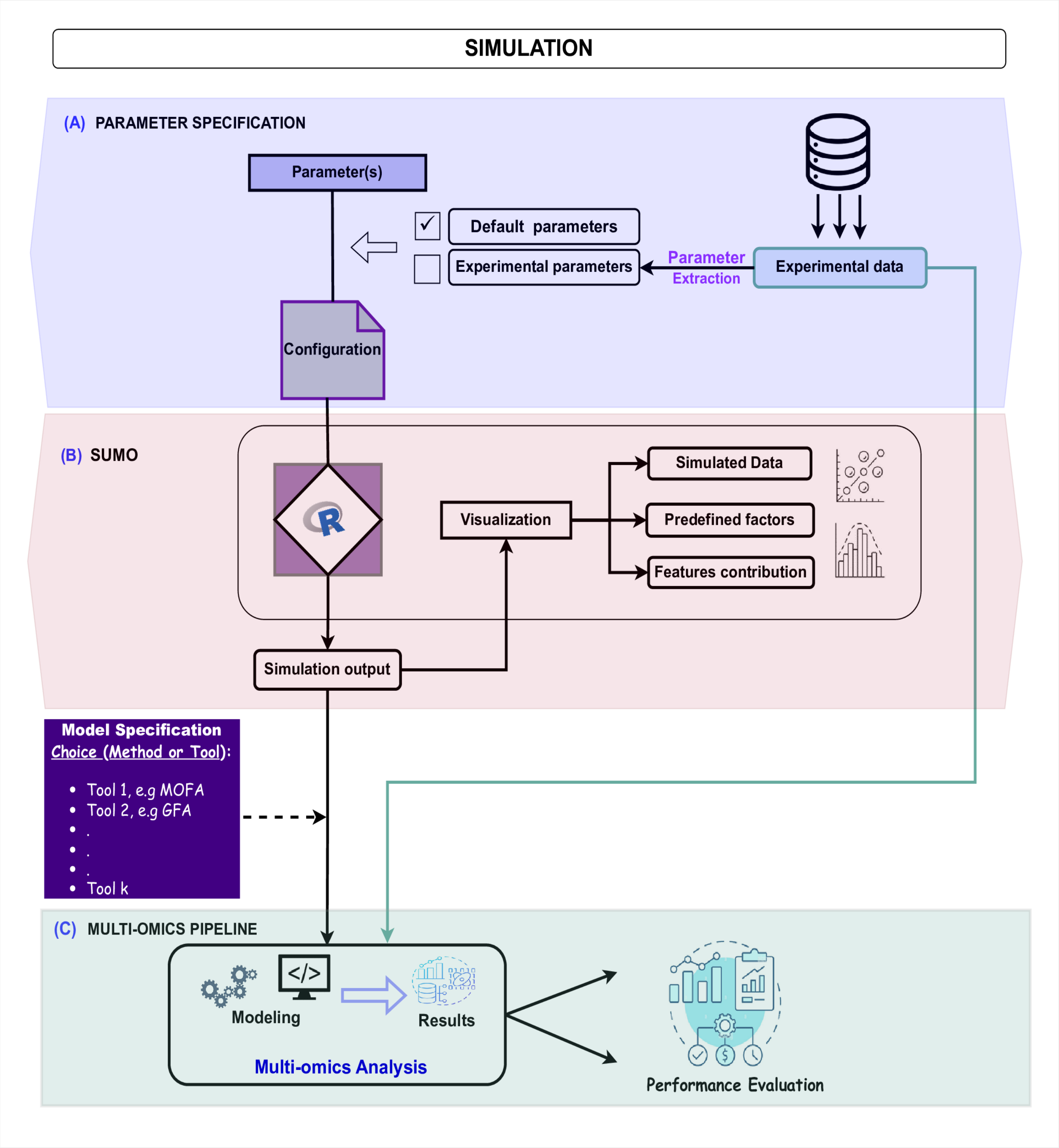

Figure S3: Detailed simulation workflow implemented in SUMO, illustrating the parameter specification phase, dataset generation, and integration with multi-omics pipelines for method evaluation

(i) **Choosing the type of signal.** The first step in configuring a SUMO simulation involves selecting the type of signal to embed within the data. Users may specify a *shared signal*, present across multiple omics layers (e.g., gene expression and proteomics), a *unique signal*, confined to a single omics dataset, or a *mixed configuration*, where both shared and unique signals coexist. This choice directly influences whether the simulated datasets capture cross-omics dependencies, modality-specific structure, or a combination of both.

(ii) **Specifying the number of factors.** SUMO allows precise control over dataset complexity through the specification of the number of latent factors. A single factor introduces one dominant signal driving the observed variation, whereas multiple factors generate richer and more intricate patterns, enabling simulation of realistic multi-factorial biological processes. The framework automatically constructs the corresponding data structures for each scenario.

(iii) **Defining feature and sample groups.** Users determine the subsets of features and samples in which the simulated signals are embedded. Selected subsets represent the "informative" regions of the data, while the remaining features and samples are modeled as uninformative variation or background noise. This design enables systematic evaluation of whether methods can distinguish true signals from irrelevant patterns.

(iv) **Adjusting signal strength and noise.** A defining aspect of SUMO is its ability to modulate the detectability of signals by tuning both signal strength and background noise. Strong signals with minimal noise represent favorable conditions, whereas weak signals embedded in high noise simulate challenging real-world scenarios. This flexibility allows rigorous testing of method robustness under diverse conditions of signal-to-noise ratios.

In SUMO, users are given precise control over the definition of latent signal characteristics by specifying the mean and variance of the sample scores and feature loadings that underpin the factor structure. These two components—loading vectors (features) and score vectors (samples)—jointly determine how signal propagates across the dataset, with their interaction forming the structured component of the data matrix. To emulate the variability encountered in real-world experiments, this structured signal is subsequently perturbed by added noise. SUMO supports two complementary strategies for parameterization:

1. **Default configuration.** By default, SUMO generates normally distributed scores and loadings with predefined means and variances, providing a straightforward entry point for users who wish to rapidly simulate synthetic datasets.

2. **Experimental data–driven configuration.** For greater biological realism, users can employ the `compute_means_var()` function to estimate empirical means and variances directly from experimental datasets. This approach allows simulated data to inherit statistical properties from real omics measurements, thereby enhancing fidelity to observed biological complexity.

Once the subsets of features and samples designated to carry signal are defined, SUMO restricts signal elevation exclusively to these groups. The specified means and variances are applied to the corresponding entries of the loading and score vectors, while all remaining entries are set to zero. This targeted assignment ensures that informative structure is confined to the chosen regions of the matrix, with all other regions representing background noise or uninformative variation.

To maximize accessibility for users across diverse expertise levels, the SUMO vignette has been expanded into a guided, hands-on tutorial (see Section 2.4.1) that enables immediate simulation using default settings while supporting gradual refinement through incremental parameter adjustment. Fundamental concepts—including the distinction between shared and unique factors, the specification of latent factor numbers, and the modulation of signal-to-noise ratios—are introduced in clear, non-technical language and reinforced with intuitive visual examples that illustrate their impact on simulated datasets. The vignette, which has been formally published and is openly accessible, functions as a fully self-contained resource, bundling executable code, test datasets, and comprehensive documentation that guides users through the acquisition, installation, and execution of SUMO. In addition, detailed explanations of each configurable parameter, including their purpose and admissible ranges, ensure that even users with limited prior experience in simulation can generate biologically meaningful datasets and confidently apply them in benchmarking and validation studies.

3.2 SUMO Data and Visualization

To illustrate SUMO's capabilities, we define a concrete example simulation setting inspired by the chronic lymphocytic leukemia (CLL) dataset, which includes multiple omics layers. For this study, we focused on two modalities—DNA methylation and mRNA expression—where integrative analysis previously identified both shared latent factors and modality-specific signals (Figure S5a). Using SUMO, we generated two datasets, $\mathbf{X_1}$ and $\mathbf{X_2}$, comprising 4,000 and 3,000 features, respectively, across 100 matched samples. The simulation was parameterized to embed one latent factor shared across both omics layers and an additional factor unique to $\mathbf{X_1}$, thus mimicking the interplay of global and dataset-specific variation. To ensure biological realism, noise and signal parameters were empirically estimated from the CLL data, and the signal-to-noise ratio (SNR) was systematically varied between 0.05 and 2.0 to produce datasets of increasing difficulty.

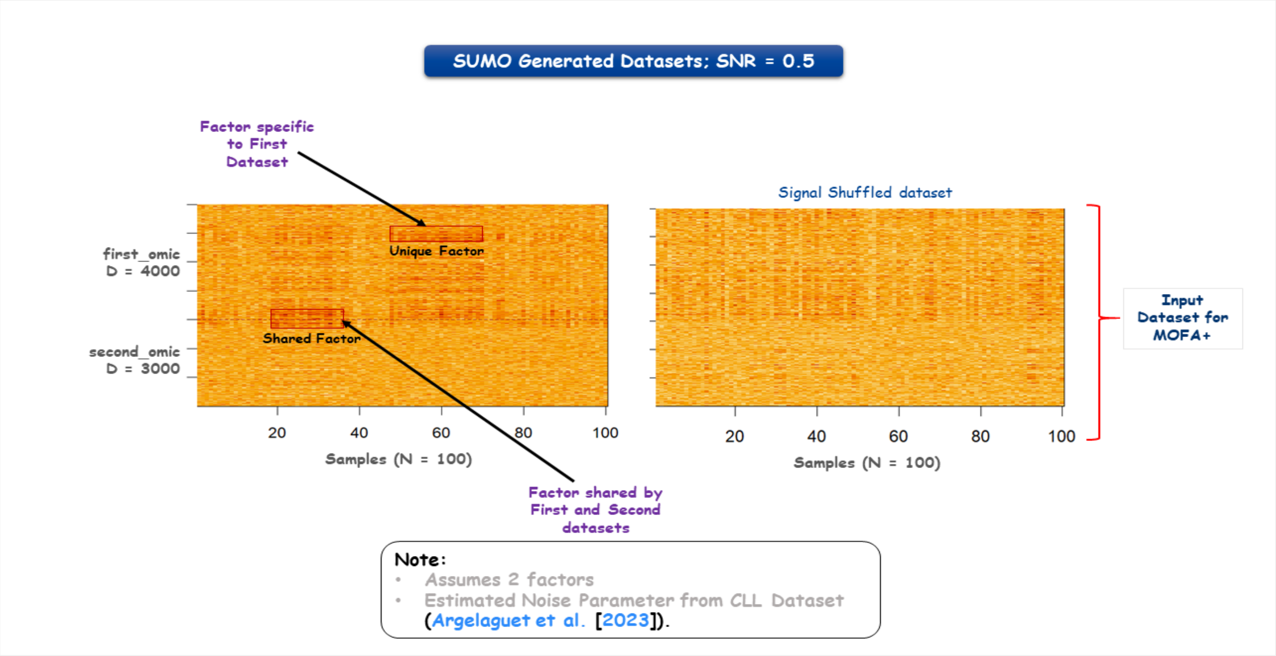

Figure S4: SUMO-generated dataset

A visual representation of one such simulated dataset is shown in Figure S4. The left panel illustrates a dataset with structured signals, where the user-defined latent factors (one shared and one unique) are clearly visible. The right panel shows the same dataset after permutation of signal regions, thereby removing block structure while preserving the underlying factor distributions. This dual representation

highlights SUMO's flexibility in generating both structured and randomized signal architectures. Importantly, the uninformative background variation against which these factors are embedded was estimated directly from the CLL dataset, ensuring that the simulated data inherit biologically realistic noise characteristics. When analyzed with MOFA (Figure S5b), the integrative model successfully recovered both the shared and dataset-specific factors, underscoring SUMO's utility as a reproducible and biologically grounded testbed for benchmarking integrative methods.

3.2.1 *Visualization*

Visualization plays a central role in demonstrating both the structure of simulated datasets and their integration into benchmarking workflows. In this study, we used SUMO to generate multi-omics datasets with user-defined latent factors (Figure S4), enabling clear inspection of the embedded signals prior to downstream analysis. These visualizations not only confirm that the simulated data faithfully reflect the specified design (e.g., shared versus unique factors), but also provide an intuitive basis for comparing structured and permuted signals. Once generated, the datasets feed directly into the benchmarking workflow (Figure S3), where they undergo model specification and integrative analysis using MOFA in our example, although SUMO is fully compatible with a wide range of factorization or clustering methods. This framework illustrates how SUMO bridges data generation and method evaluation: simulated datasets are first visualized and validated, then seamlessly integrated into benchmarking pipelines. The outcomes of this process, including recovery of shared and modality-specific factors, are reported in the main paper (see Section 3), underscoring SUMO's role as a reproducible and biologically grounded simulation platform for method evaluation.

3.3 Performance Evaluation Environment

To demonstrate the practical utility of SUMO within an integrative analysis workflow, we employed MOFA as a well-established reference framework for multi-omics factorization. MOFA was applied both to a real-world chronic lymphocytic leukemia (CLL) dataset [Argelaguet et al., 2023] and to SUMO-generated datasets, enabling direct comparison between biologically derived and synthetically controlled

data.

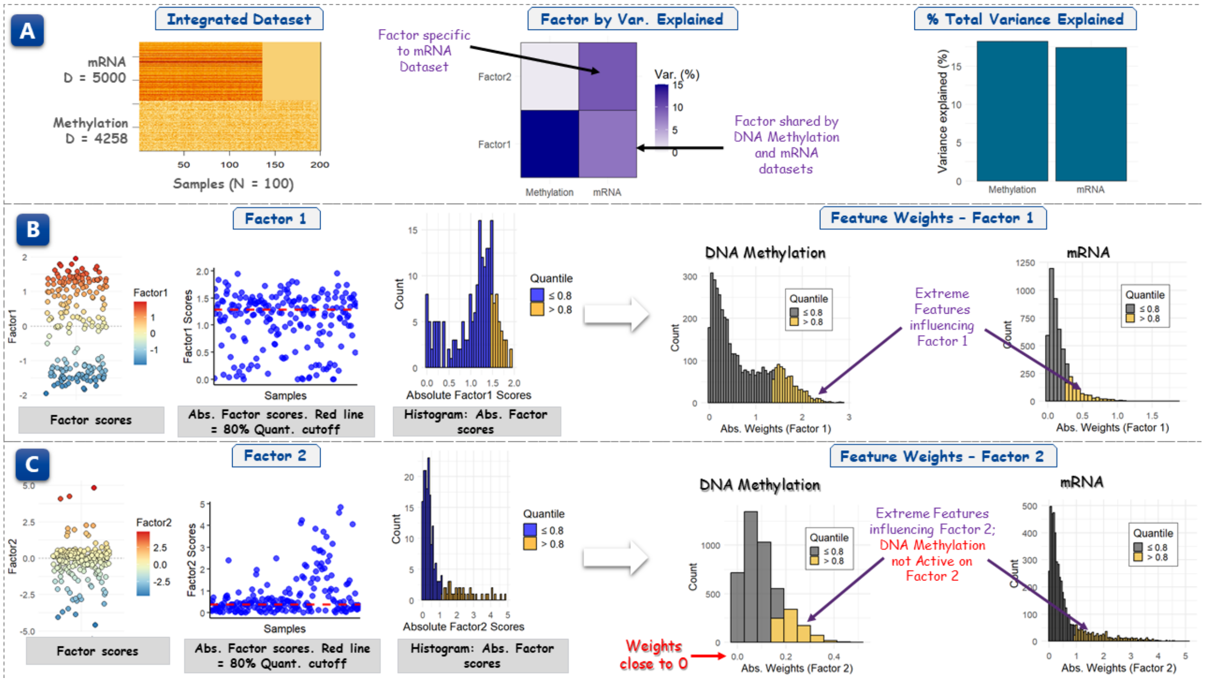

(a) *Integrated Analysis using MOFA; (A) Overview of the integrated dataset containing mRNA and DNA methylation features with factor decomposition illustrating shared and dataset-specific contributions. (B) Factor 1 captures variability influenced by both mRNA and DNA methylation, highlighting extreme feature weights. (C) Factor 2 is predominantly driven by mRNA, with DNA methylation features showing negligible influence.*

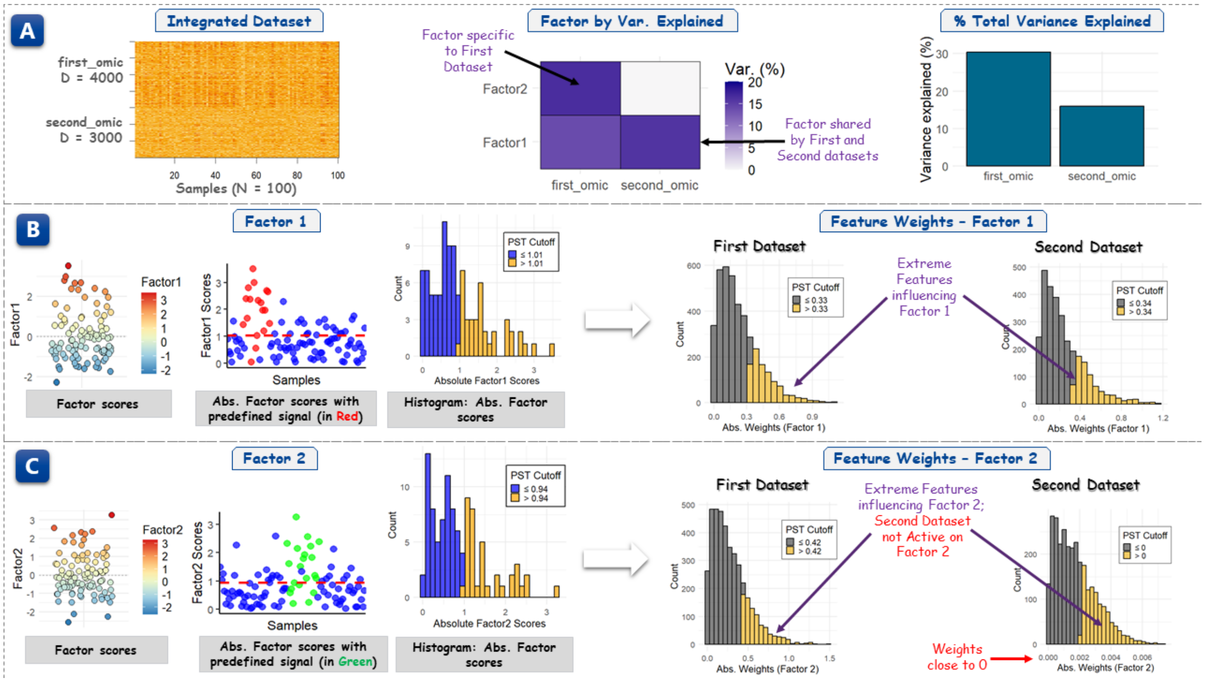

(b) *Simulation Data Analysis; (A) SUMO-generated simulated multi-omics dataset. (B & C) Factor decomposition of the simulated dataset highlights the structure imposed during simulation. PST cutoff - Peak-to-spread cutoff.*

Figure S5: Overview of Multi-Omics Analysis: Panel A shows the integrated analysis of real-world dataset using MOFA and Panel B illustrates the simulation data analysis and integration using MOFA.

Further, we simulated dataset based on the CLL study where we estimated the mean and variability of the feature loadings and the factor scores which are the main component in data generation model. We also estimated the background noise using different assumed valued for signal to noise ratios (SNR) in this case low SNRs (0.05), medium SNRs (0.5 & 1.0), and high SNRs (1.5 & 2.0). The main objective is to demonstrate how developers can assess their method robustness over varying noisy levels and track the behaviour of their method.

The visual outputs presented in Figure S6a provide compelling evidence of MOFA's capacity to disentangle structured signal from background noise under varying signal-to-noise ratio (SNR) conditions. As the SNR increases from 0.05 to 2.0, there is a noticeable improvement in the clarity and definition of patterns within the heatmaps of the input datasets, reflecting enhanced detectability of underlying latent structures. This trend is further reinforced by the factor score scatter plots, where the separation between signal and noise becomes progressively more distinct. At low SNR levels, MOFA struggles to clearly differentiate signal from noise, as indicated by the overlapping scatter patterns. However, as the SNR improves, the factor scores corresponding to the signal form well-defined clusters, illustrating the model's improved ability to recover meaningful biological variation. Correspondingly, the bar plots depicting factor contributions show a growing proportion of variance being attributed to biologically relevant factors, and a simultaneous decline in noise-driven components, reinforcing the notion that higher SNR supports more accurate factor recovery across omics layers.

Graphically, this behavior is quantitatively captured in Figure S6b, which plots the relationship between SNR and both variance explained and noise contribution across two omics datasets. As the SNR increases, the proportion of variance explained by MOFA rises sharply for both datasets, plateauing at higher SNR levels, indicative of stable and reliable factor estimation. Simultaneously, the contribution of noise decreases markedly, underscoring a reduced influence of random variation in shaping latent structure. This inverse relationship between signal fidelity and noise interference highlights the importance of high-quality input datasets for successful integration and interpretation in multi-omics analyses. Collectively, these visual and graphical findings underscore SUMO's utility in generating benchmark datasets with controllable noise properties, and MOFA's effectiveness in recovering latent factors under favorable

signal conditions.

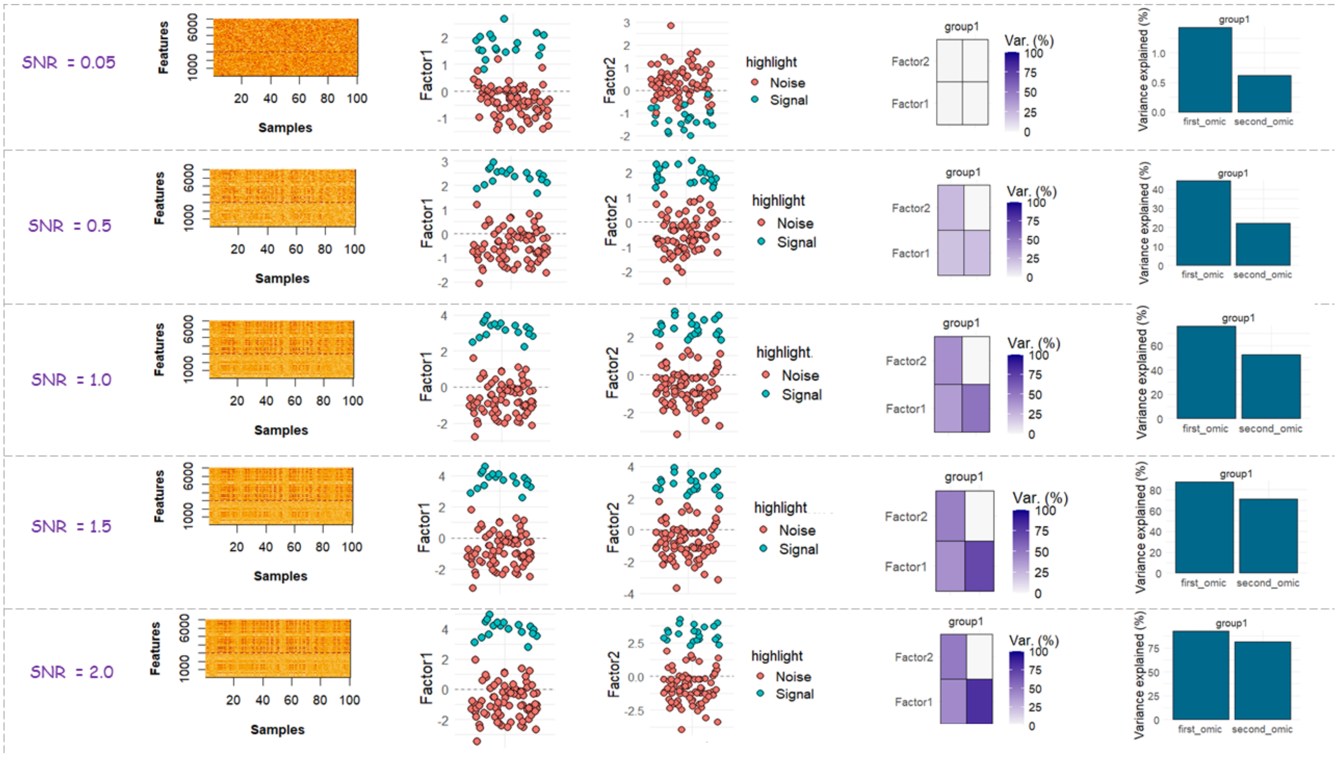

(a) *MOFA Results Across Different Signal-to-Noise Ratios (SNRs): Visualization of MOFA-derived factorization across datasets simulated with varying SNR levels (0.05–2.0). Each row corresponds to a different SNR condition, showing (from left to right): heatmaps of input data, factor score scatter plots distinguishing signal from noise, factor contributions, and variance explained across omics layers. As SNR increases, the separation between signal and noise becomes more distinct, and the variance explained by factors stabilizes across omics layers, highlighting the impact of noise levels on latent factor discovery.*

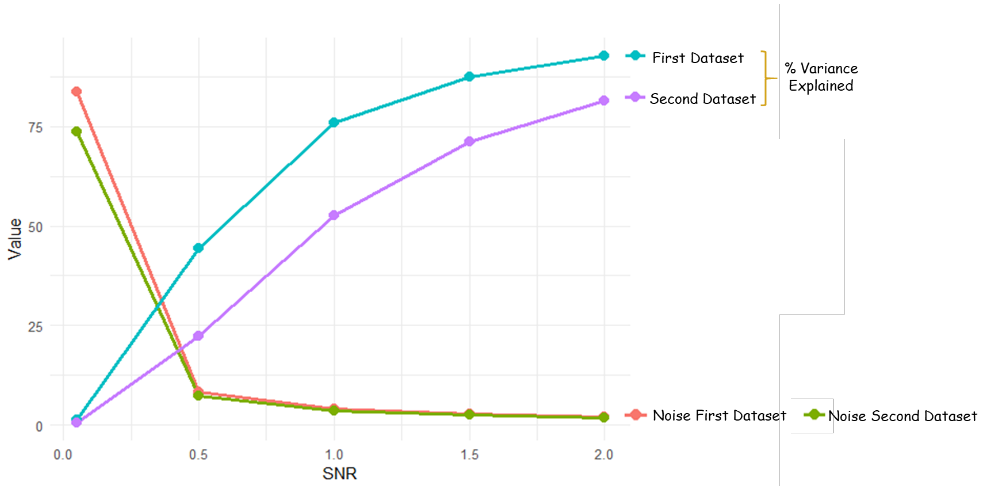

(b) *Effect of Signal-to-Noise Ratio (SNR) on Variance Explained and Noise Contribution: This plot illustrates the relationship between SNR and the proportion of variance explained across two datasets. As SNR increases, the variance explained by the first (cyan) and second (purple) datasets rises, while the noise contribution in both datasets (red and green) decreases sharply and stabilizes at higher SNR levels. This trend highlights how increasing signal strength enhances data interpretability while reducing noise interference.*

Figure S6: Panel A shows how MOFA factorization separates signal from noise across datasets with increasing SNR. Panel B quantifies this effect, revealing how higher SNR boosts variance explained while minimizing noise interference.

3.4 Structural resemblance of SUMO-generated data to the real-experimental data

An important consideration when simulating multi-omics data is the extent to which the generated datasets resemble real experimental data. SUMO has been designed to approximate structural properties observed in experimental omics data while maintaining flexibility for benchmarking purposes. Specifically, SUMO preserves key characteristics such as sample-wise variance, feature distributional heterogeneity, and controlled levels of signal-to-noise ratio (SNR). To achieve this, the simulator uses the overall variance from real data as a proxy for background noise, upon which biologically interpretable latent factors are superimposed. This approach ensures that the simulated datasets retain realistic levels of stochastic variability while also containing clearly defined signals that can be systematically tuned.

It is important to emphasize that the objective of SUMO is not to reproduce real datasets exactly, but rather to provide datasets that are structurally comparable to real-world omics data. By controlling the strength, distribution, and overlap of latent factors across different omics layers, SUMO allows users to generate scenarios that mimic core features of real data while still enabling transparent benchmarking of integrative methods. As such, SUMO bridges the gap between purely synthetic simulations, which often lack realism, and the complexity of experimental datasets, which may lack ground truth.

To illustrate this resemblance, we compared SUMO-generated datasets with CLL data used in our demonstrations. Both data types displayed comparable distributions of variance across features and similar clustering patterns of samples, indicating that SUMO realistically captures the global structure of multi-omics data. Importantly, SUMO achieves this while offering explicit ground truth latent factors, which are not available in real data. This dual property of realism and controllability makes SUMO particularly useful for testing, validating, and comparing factor-based and other integrative approaches in multi-omics research.

3.4.1 Compare Principal Components (PC) trends

One way to illustrate this structural resemblance is by comparing the principal components (PCs) of real and SUMO-generated datasets. As shown in Supplementary Figure S7, both data types exhibit a strong concentration of variance in the first one or two PCs, followed by a sharp drop-off in explained variance across subsequent components. For Omic1, the first PC alone captures the majority of the variance in both real and simulated data, while for Omic2, the variance is distributed across the first two PCs in a similar manner.

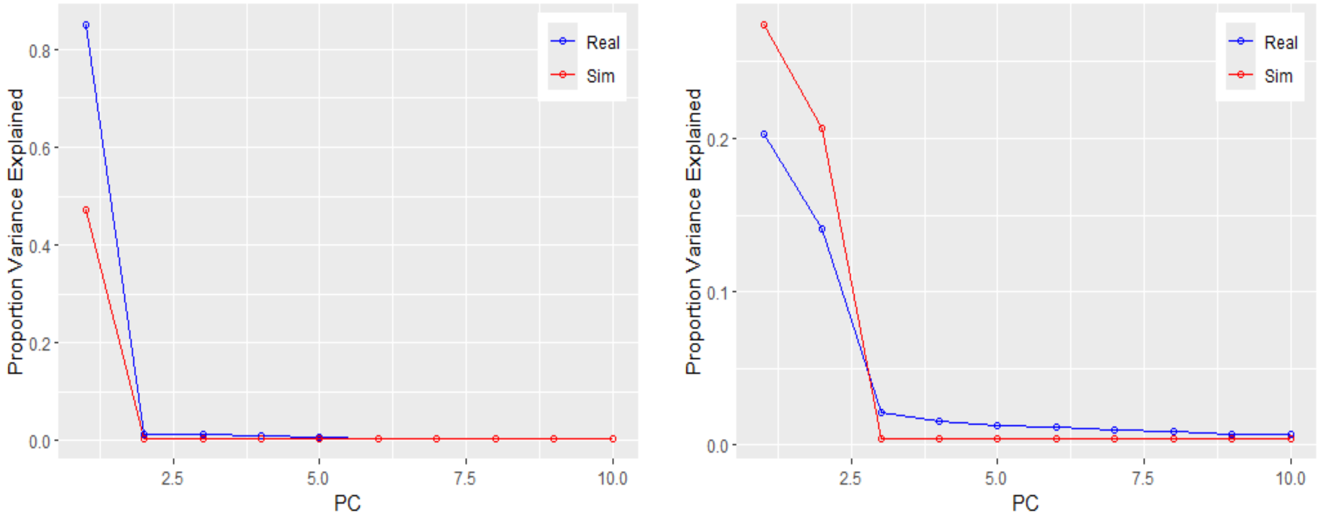

(a) PC variance (Real dataset)

(b) PC variance (Simulated dataset)

Figure S7: Variance explained by top PCs in real vs. SUMO data. Both show 1–2 dominant PCs, indicating similar low-dimensional structure.

The cumulative variance plots (Supplementary Figure S8) confirm this trend, with simulated datasets closely tracking the trajectory of real datasets. This indicates that SUMO realistically reproduces the low-dimensional structure typical of multi-omics datasets, where a small number of latent factors dominate the observed variation.

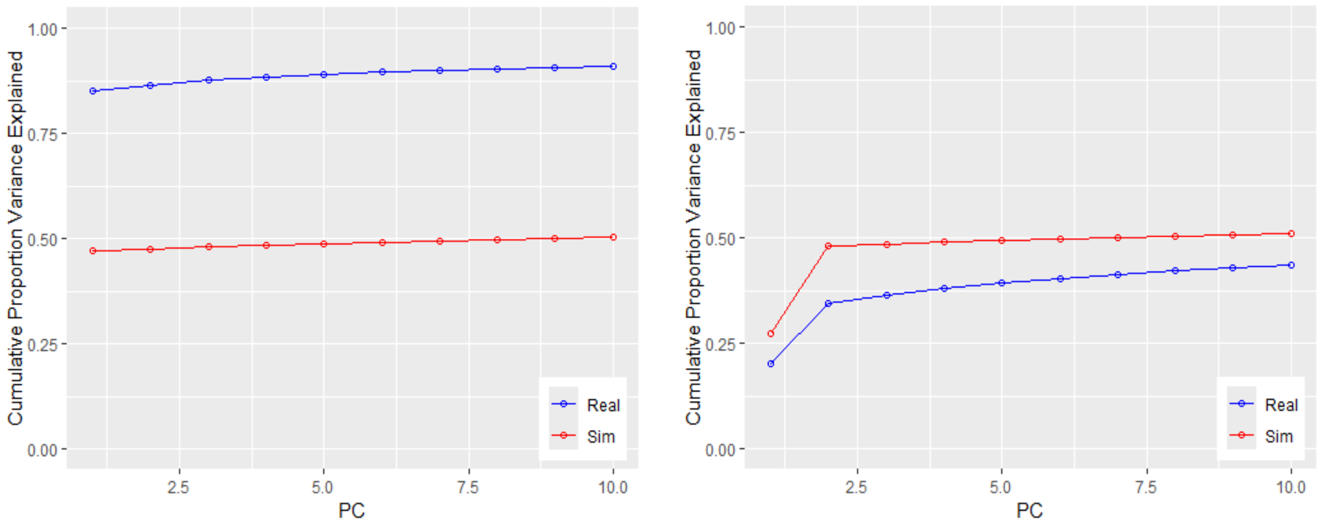

(a) Cumulative PC variance (Real dataset) (b) Cumulative PC variance (Simulated dataset)

Figure S8: Cumulative variance explained by PCs in real vs. SUMO data. Simulated datasets follow the same trajectory as real data.

3.4.2 Compare the correlations distributions

To further assess the structural resemblance, we compared the correlation distributions of features in real and SUMO-generated datasets (Supplementary Figure S9). For Omic1, the real dataset exhibits a clear peak of strong positive correlations (furthest from zero), indicating the presence of tightly co-expressed feature groups. The SUMO-generated dataset shows a more centralized correlation distribution, with a smaller secondary peak near high correlations. This reflects the fact that SUMO preserves the global correlation structure but introduces controlled signal blocks rather than reproducing all complex dependencies present in the real data.

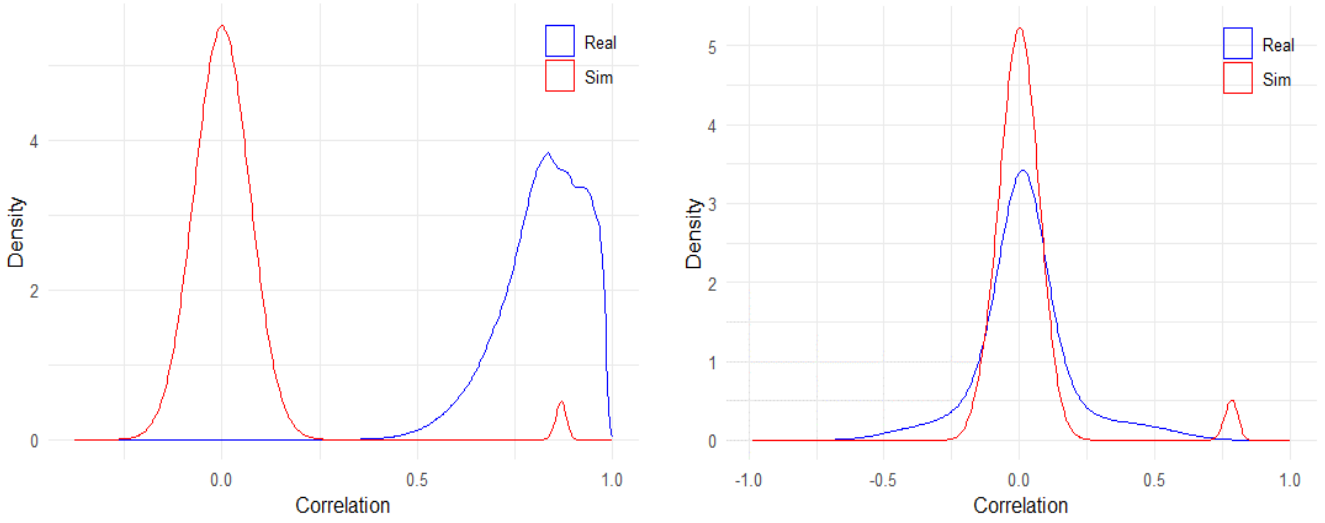

(a) Correlation density (Real dataset)   (b) Correlation density (Simulated dataset)

Figure S9: Illustration of the simulation setting for an overlapping cluster in which the similarity is induced for two coinciding sets of compounds. Panel a: Data set D1. Panel b: Data set D2.

For Omic2, both real and SUMO-generated datasets display a similar overall trend, with the majority of feature correlations centered near zero and only a small fraction showing moderate to strong correlation. The similarity in correlation density between real and simulated data highlights that SUMO captures the broad structural properties of multi-omics datasets. The slight differences are intentional: SUMO adds an additional layer of latent signal designed to be recovered by factorization methods. This ensures that while the simulated data resemble real omics datasets in their overall correlation structure, they remain sufficiently controlled to allow clear benchmarking of integrative analysis tools.

3.4.3 Exploration of the feature-level variances

We also compared the variance distributions of features between real and SUMO-generated datasets (Supplementary Figure S10). For both Omic1 and Omic2, the simulated datasets were parameterized using the empirical variance from real data, ensuring that the central tendency of feature-level variance (median and mean) is preserved. This alignment highlights that SUMO realistically captures the noise scale of experimental omics datasets.

At the same time, some deviations are expected and indeed desirable. In Omic1, the real data contain a subset of highly variable features, while the simulated dataset shows a tighter variance distribution

with controlled spread. For Omic2, the real and simulated distributions overlap closely at the median, although SUMO exhibits an extended upper tail reflecting the injected signal. These differences arise because SUMO superimposes structured latent signals onto the noise baseline, leading to additional variance components that are absent from the purely observational real dataset. This property ensures that while SUMO preserves realistic noise characteristics, it also introduces identifiable signals that can be systematically recovered by integrative factorization methods.

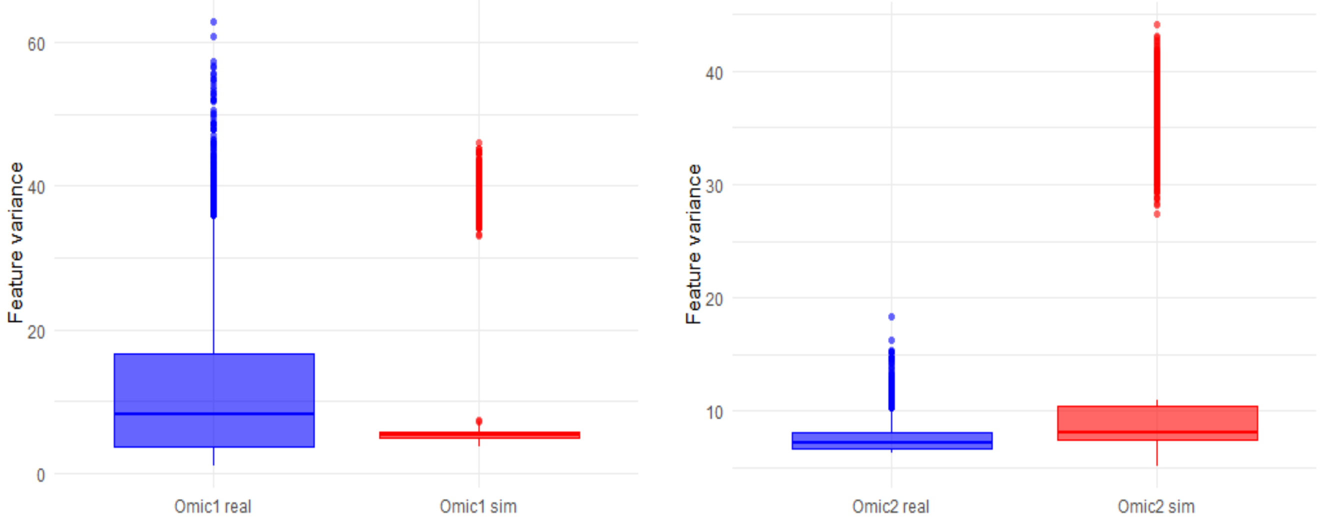

(a) Feature variance (Real dataset)　　(b) Feature variance (Simulated dataset)

Figure S10: Feature variance comparison between real and SUMO data. Medians align closely; deviations reflect added signals.

3.5 Compare Clusters using Silhoutte Profiles

Finally, we compared clustering structures between real and SUMO-generated datasets using silhouette profiles across different numbers of clusters (Supplementary Figure S11). The average silhouette width across all samples indicates the overall clarity of the clustering structure, with higher values reflecting more compact and well-separated clusters. By examining silhouette profiles across different numbers of clusters, one can identify the most likely underlying cluster structure in the data and assess whether the observed groupings are robust or diffuse. In both cases, the general trend is preserved: average silhouette width is highest for small numbers of clusters (particularly $k = 2$) and decreases as k increases. This demonstrates that the underlying global cluster tendency is captured by SUMO, even though absolute

silhouette values may differ between real and simulated data.

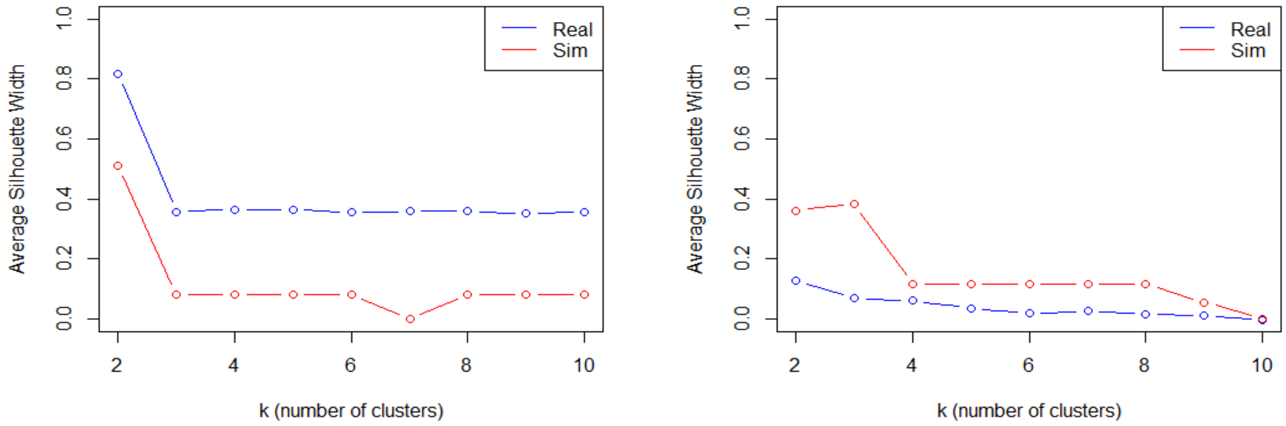

(a) Silhouette profiles (Real dataset)

(b) Silhouette profiles (Simulated dataset)

Figure S11: Silhouette profiles for real and SUMO data. Both show strongest clustering at $k = 2$, with similar declining trends.

The observed differences are expected and can be attributed to the controlled addition of latent signals and noise in the simulation. While the real datasets reflect complex biological variability, the simulated datasets incorporate structured latent factors that can weaken average silhouette values but still preserve the main clustering patterns. Importantly, the fact that both real and SUMO datasets highlight the presence of similar dominant clusters confirms that SUMO reproduces realistic clustering tendencies while also embedding benchmarkable signals for recovery by factorization methods.

// Acknowledgments section
4 Acknowledgments

Conflict

All authors have no conflict of interest to declare

Ethics and Transparency

We utilized a generative language model to refine the language of the manuscript. The model was specifically applied to correct grammar, improve readability, bringing clarity across sections. The model was not used to generate any original scientific content, methodologies, or results presented in this paper, ensuring the integrity of our scientific findings.

References

Ricard Argelaguet, Britta Velten, Damien Arnol, Sascha Dietrich, Thorsten Zenz, John C Marioni, Florian Buettner, Wolfgang Huber, and Oliver Stegle. Multi-omics factor analysis—a framework for unsupervised integration of multi-omics data sets. Molecular systems biology, 14(6):e8124, 2018.

Ricard Argelaguet, Britta Velten, Damien Arnol, Florian Buettner, Wolfgang Huber, and Oliver Stegle. MOFAdata: Data package for Multi-Omics Factor Analysis (MOFA), 2023. URL https://bioconductor.org/packages/MOFAdata. R package version 1.18.0.

Baptiste Auguie, Anton Antonov, and Maintainer Baptiste Auguie. Package 'gridextra'. Miscellaneous functions for "grid" graphics, 9, 2017.

Brielin C Brown, Collin Wang, Silva Kasela, François Aguet, Daniel C Nachun, Kent D Taylor, Russell P Tracy, Peter Durda, Yongmei Liu, W Craig Johnson, et al. Multiset correlation and factor analysis enables exploration of multi-omics data. Cell Genomics, 3(8), 2023.

Yehudit Hasin, Marcus Seldin, and Aldons Lusis. Multi-omics approaches to disease. Genome biology, 18:1–15, 2017.

Sepp Hochreiter, Ulrich Bodenhofer, Martin Heusel, Andreas Mayr, Andreas Mitterecker, Adetayo Kasim, Tatsiana Khamiakova, Suzy Van Sanden, Dan Lin, Willem Talloen, et al. Fabia: factor analysis for bicluster acquisition. Bioinformatics, 26(12):1520–1527, 2010.

R Core Team. R: A Language and Environment for Statistical Computing. R Foundation for Statistical Computing, Vienna, Austria, 2013. URL http://www.R-project.org/. ISBN 3-900051-07-0.

Hadley Wickham, Winston Chang, and Maintainer Hadley Wickham. Package 'ggplot2'. Create elegant data visualisations using the grammar of graphics. Version, 2(1):1–189, 2016.
